# The impact of exposure to shift-based schedules on medical students

**DOI:** 10.3402/meo.v20.27434

**Published:** 2015-06-22

**Authors:** David A. Williams, Jennifer R. Kogan, Karen E. Hauer, Traci Yamashita, Eva M. Aagaard

**Affiliations:** 1 Denver Veterans Affairs Medical Center, Department of Internal Medicine, University of Colorado School of Medicine, Aurora, CO, USA; 2 Perleman School of Medicine, Department of Medicine, University of Pennsylvania, Philadelphia, PA, USA; 3 Department of Medicine, University of California San Francisco School of Medicine, San Francisco, CA, USA; 4 Department of Medicine, University of Colorado School of Medicine, Aurora, CO, USA

**Keywords:** duty-hour reform, shift-based schedules, medical students, internal medicine clerkship, team fragmentation

## Abstract

**Background:**

With new resident duty-hour regulations, resident work schedules have progressively transitioned towards shift-based systems, sometimes resulting in increased team fragmentation. We hypothesized that exposure to shift-based schedules and subsequent team fragmentation would negatively affect medical student experiences during their third-year internal medicine clerkship.

**Design:**

As part of a larger national study on duty-hour reform, 67 of 150 eligible third-year medical students completed surveys about career choice, teaching and supervision, assessment, patient care, well-being, and attractiveness of a career in internal medicine after completing their internal medicine clerkship. Students who rotated to hospitals with shift-based systems were compared to those who did not. Non-demographic variables used a five-point Likert scale. Chi-squared and Fisher's exact tests were used to assess the relationships between exposure to shift-based schedules and student responses. Questions with univariate *p*≤0.1 were included in multivariable logistic regression models.

**Results:**

Thirty-six students (54%) were exposed to shift-based schedules. Students exposed to shift-based schedules were less likely to perceive that their attendings were committed to teaching (odds ratio [OR] 0.35, 95% confidence interval [CI]: 0.13–0.90, *p*=0.01) or perceive that residents had sufficient exposure to assess their performance (OR 0.29, 95% CI: 0.09–0.91, *p*=0.03). However, those students were more likely to feel their interns were able to observe them at the bedside (OR 1.89, 95% CI: 1.08–3.13, *p*=0.02) and had sufficient exposure to assess their performance (OR 3.00, 95% CI: 1.01–8.86, *p*=0.05).

**Conclusions:**

These findings suggest that shift-based schedules designed in response to duty-hour reform may have important broader implications for the teaching environment.

The student–teacher relationship is at the core of medical education, and effective relationships are critical for achieving favorable learning outcomes for medical students (1–4). Such relationships take time to develop, and studies suggest that medical students’ clinical experiences may be enhanced by deliberately structuring longitudinal attachments to supervisors (2, 5).

However, schedule alterations, most recently in response to Accreditation Council for Graduate Medical Education (ACGME) duty-hour regulations implemented in July 2011, may lead to an increase in team fragmentation and all too often come at the expense of team continuity (2, 6–9). Such discontinuity begets inefficiency and lack of connection, leading to great frustration and anxiety in learners and incredible challenges for teachers (10, 11). At the University of Colorado School of Medicine (UCSOM), third-year medical students on the inpatient internal medicine clerkship rotate through two of six possible sites, which include a university hospital, a large public hospital, a veterans affairs hospital, and three community hospitals. Inpatient teams are structured as follows: university: 1 attending, 1 resident, 1 intern, and 1 student; public hospital: 1 attending, 1 resident, 1 intern, and 1 student; veterans affairs hospital: 1 attending, 1 resident, 2 interns, and 2 students; and community hospitals: 1 attending; 0–1 resident; 0–2 interns; and 1–2 students. For many years, the public hospital has utilized a shift-based system wherein residents rotate among day, swing (5–11 pm), and night shifts. In July 2012, in response to duty-hour reform, the university hospital restructured resident rotations to adopt a similar schedule. At both sites, medical students stay on the day shift and do not rotate with the residents as they move between shift types. The other four hospitals have continued to utilize a night float or an overnight call system with consistent pairing of students and residents during the day shifts.

We hypothesized that exposure to shift-based schedules with rotating residents would influence student supervision, feedback, teaching, and well-being, through the increase in team fragmentation, thereby leading to a worse medical student educational experience.

## Methods

A cross-sectional, 46-item survey was performed at the UCSOM as part of *The effect of resident duty-hours restrictions on internal medicine clerkship experiences: surveys of medical students and clerkship directors*. All third-year medical students on internal medicine rotations during the study periods of March to June 2011 (*N*=75) and March to June 2012 (*N*=75) received an invitation via email explaining the purpose of the study and a link to the online survey. Participation was voluntary. Non-respondents received up to two additional email invitations to participate. Respondent identifying information was removed prior to analysis.

Students were considered exposed to the shift-based model of training if at least one of the two sites at which they rotated during their inpatient internal medicine rotation had adopted a shift-based schedule at the time of their rotation.

Survey items were grouped into categories measuring student perspectives on teaching/supervision, assessment, percentage of patients already worked up, team support, patient care, and well-being. They were measured using a Likert scale, unless otherwise noted, ranging from 1 to 5 representing strongly disagree, disagree, neutral, agree, or strongly agree.

Demographic characteristics are reported according to exposure groups as percentages for dichotomous characteristics and as mean with standard deviation for age at the time of survey completion. Interest in an internal medicine career was defined by at least one of five possible career choices identified including internal medicine, medicine–pediatrics, medicine–dermatology, medicine–psychiatry, or medicine–genetics. Likert item responses are reported according to exposure groups as medians [interquartile ranges].

Differences according to exposure groups were tested with two-sided chi-square tests and unpaired *t*-tests. As needed, items were reverse coded so that higher values represented more favorable responses.

For each of the categories, separate logistic regression models were used to assess the relationship between a shift-based schedule (outcome) and the measures of interest (predictors). Univariate differences in the ordinal Likert responses according to exposure were assessed with Cochran–Mantel–Haenszel tests. Items with univariate *p*≤0.1 were included as potential covariates in the logistic regression model. For some items measuring teaching/supervision and assessment, the students assessed their attendings, residents and interns separately. If any of the attending, resident, or intern components of an item had a univariate *p*≤0.1, the item and all of its components were included as potential covariates in the logistic regression model. Backward selection methods were used to determine final models when multiple potential covariates were identified. Pearson correlation coefficients were used to determine correlation between items found to be statistically significant between groups. Statistical analyses were carried out using SAS version 9.3 (SAS Institute, Cary, NC).

A reverse power calculation indicated that given 30 subjects per group, it would be possible to detect an absolute difference of about 30% between the two groups with a power of more than 80% (12–15).

The Colorado Multiple Institutional Review Board approved the study.

## Results

Sixty-seven (44.7%) of 150 eligible third-year medical students responded. Demographics were consistent with those of the class overall. Thirty-seven students (54%) were exposed to shift-based schedules, while 31 students (46%) were not. Two students, both in the group exposed to shift-based schedules, only completed the demographic data portion of the survey and were, therefore, only included in the demographics.

More students were exposed to shift-based schedules during Spring 2012 due to the university site adopting such a schedule (Table 1). Overall, greater than 90% reported that they strongly agreed or agreed that they were satisfied with the clerkship, and 66% expressed interest in internal medicine or a combined specialty with internal medicine. These variables did not vary by exposure.

**Table 1:** Demographic data

	Exposed (*n*=36)	Not exposed (*n*=31)	*p*
Age mean (SD)	26.5 (2.3)	26.3 (2.5)	0.76
Gender: female, *N* (%)	19 (52.8)	12 (38.7)	0.25
March to June 2011, *N* (%)	13 (36.1)	21 (67.7)	0.01
March to June 2012, *N* (%)	23 (63.9)	10 (32.3)	0.01
Interest in career in internal medicine	27 (75.0)	17 (54.8)	0.08

Univariate analysis yielded six survey items with a *p*≤0.1. These items and all of their components were included as potential covariates in the logistic regression model.

### Teaching and supervision

In response to the question, ‘My attendings were committed to teaching me’, the median response of exposed students was 4 with an interquartile range of [4,5], compared with 5 [4,5] for not exposed students (Table 2). Logistic regression analysis showed a statistically significant odds ratio (OR) of 0.35 with 95% confidence interval (CI) of 0.13–0.90 (*p*=0.01) (Table 3). Responses to similar questions involving interns and residents did not significantly vary by exposure. However, students exposed to shift-based schedules were statistically more likely to perceive that interns were able to observe them at the bedside (4 [4,5] vs. 4 [2,4], OR 1.89, 95% CI 1.08–3.13, *p*=0.02; Table 3). Notably, few students in both the exposed and not exposed groups felt that their attendings were able to observe them at the bedside (3[2,3] vs. 3 [2,4]), a difference which did not vary by exposure.

**Table 2:** Complete survey results prior to logistic regression analysis^a^

Category	Question	Overall (*N*=65)	Exposed (*N*=34)	Not exposed (*N*=31)	*p*
Teaching and supervision	Time to teach				
	Attendings	4 [4, 5]	4 [3, 4]	4 [4, 5]	0.11
	Residents	4 [3, 4]	4 [3, 4]	4 [3, 4]	0.89
	Interns	4 [2, 4]	4 [3, 4]	4 [2, 4]	0.77
	Able to observe at bedside				
	Attendings	3 [2, 4]	3 [2, 3]	3 [2, 4]	0.13
	Residents	4 [3, 4]	4 [3, 4]	4 [3, 5]	1.0
	Interns^b^	4 [3, 4]	4 [4, 5]	4 [2, 4]	0.04
	Effective teaching				
	Attendings^b^	4 [4, 5]	4 [4, 5]	5 [4, 5]	0.02
	Residents^b^	4 [4, 5]	4 [4, 5]	4 [4, 5]	0.10
	Interns	4 [4, 5]	4 [4, 5]	4 [3, 5]	0.43
	Committed to teaching				
	Attendings^b^	*N*=64, 5 [4, 5]	4 [4, 5]	*N*=30, 5 [4, 5]	0.04
	Residents	5 [4, 5]	4.5 [4, 5]	5 [4, 5]	0.70
	Interns	4 [4, 5]	4 [4, 5]	4 [4, 5]	0.41
	Appropriately supervised				
	Attendings	*N*=64, 4 [3, 4]	4 [3, 4]	*N*=30, 4 [3, 5]	0.15
	Residents	*N*=64, 4 [4, 5]	4 [4, 5]	*N*=30, 55 [4, 5]	0.38
	Interns	4 [4, 5]	4 [4, 5]	4 [4, 5]	0.82
	Rotation enabled me to learn internal medicine	5 [4, 5]	4 [4, 5]	5 [4, 5]	0.59
	I feel adequately prepared for my sub-I	*N*=62, 4 [4, 5]	*N*=33, 4 [4, 5]	*N*=29, 4 [4, 5]	0.52
	Overall satisfaction	5 [4, 5]	4 [4, 5]	5 [4, 5]	0.41
Assessment	Useful feedback				
	Attendings	4 [4, 5]	4 [4, 4]	4 [4, 5]	0.11
	Residents	4 [4, 5]	4 [4, 5]	4 [4, 5]	0.58
	Interns	4 [4, 5]	4 [4, 5]	5 [4, 5]	0.61
	Had sufficient exposure to me to evaluate my performance				
	Attendings^b^	4 [3, 4]	4 [3, 4]	4 [3, 5]	0.06
	Residents	4 [4, 5]	4 [4, 5]	5 [4, 5]	0.30
	Interns	5 [4, 5]	4.5 [4, 5]	5 [4, 5]	0.49
Patient care	Satisfaction with care able to deliver	4 [4, 4]	4 [4, 4]	4 [4, 4]	0.37
	Able to follow a patient throughout their hospitalization	4 [4, 5]	4 [4, 5]	5 [4, 5]	0.30
	The care of patients on this service was fragmented^c^	3 [2, 4]	3 [2, 4]	3 [2, 4]	0.38
	Residents appeared satisfied with the care they deliver	4 [3, 4]	4 [3, 4]	4 [3, 4]	0.93
Well-being	Established meaningful relationship with team members	4 [4, 5]	4 [4, 5]	5 [4, 5]	0.31
	Felt supported by my team^b^	4 [4, 5]	4 [4, 5]	5 [4, 5]	0.08

Distribution of five-point Likert questions: 1=strongly disagree, 2=disagree, 3=neutral, 4=agree, 5=strongly agree.

a The values are reported as median [interquartile range].

b Included in logistic regression model.

c Reverse coded: 1=strongly agree; 2=agree; 3=neutral, 4=disagree; 5=strongly disagree.

Students exposed to shift-based schedules were less likely to perceive their attending provided effective teaching (4 [4,5] vs. 5 [4,5], OR 0.34, 95% CI 0.14–0.81, *p*=0.01); however, this question was highly correlated with the question regarding attending commitment to teaching by Pearson correlation coefficients, thus bringing the significance of this finding into question.

**Table 3:** Logistic regression model with statistically significant results

Question	OR^a^	95% Confidence interval	*p*
Attendings committed to teaching	0.35	0.13–0.90	0.01
Interns observe at the bedside	1.89	1.08–3.13	0.02
Interns assess student performance	3.00	1.01–8.86	0.05
Residents assess student performance	0.29	0.09–0.91	0.03
Meaningful relationships	0.51	0.24–1.11	0.09

a Positive odds ratio (OR) favors exposure.

### Assessment

Exposed students perceived that residents were statistically less likely to have sufficient contact to assess their performance (OR 0.29, 95% CI: 0.09–0.91, *p*=0.03; Table 3), but felt that interns were more likely to have sufficient exposure to them to assess their performance (OR 3.00, 95% CI: 1.01–8.86, *p*=0.05; Table 3). The percentage of students reporting their attendings having sufficient exposure to them to assess their performance was low in both exposed (4 [3,4]) and not exposed (4 [3,5]) groups, although the difference did not vary between groups.

### Patient care

**Fig. 1:**
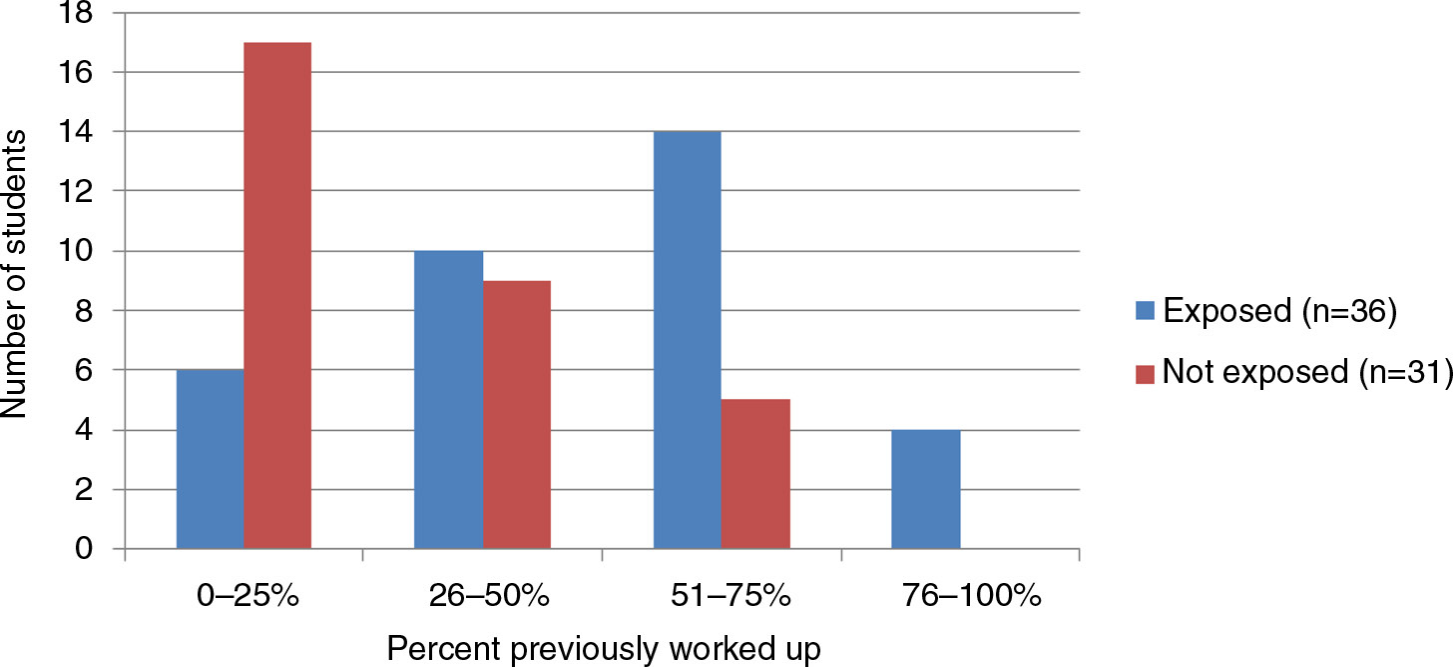
Percent of patients admitted who were previously worked up.

Students in the exposed groups were significantly more likely to report admitting patients who were previously worked up (OR 3.10, 95% CI: 1.62–5.96, *p*≤0.001; Fig. 1). However, there was no variation by exposure with regard to perceived satisfaction with the patient care students were able to deliver, ability to follow a patient through their hospitalization, or patient care fragmentation.

### Well-being

There was a non-statistically significant trend towards students feeling less team support in the exposed group (OR 0.51, 95% CI: 0.24–1.11, *p*=0.09; Table 3). There was no difference in perceived establishment of meaningful relationships with team members.

## Discussion

Although duty-hour regulations are here to stay, schedule modifications to comply with these regulations remain largely up to individual programs (9). As residency programs adjust resident schedules, the impact on medical students should be considered. In our survey, we found that exposure to shift-based schedules decreased student perception of attendings’ commitment to teaching, decreased student perception of residents’ ability to assess student performance, increased student perception of interns’ ability to assess student performance and observe them at the bedside, and increased student perception of admitting previously worked-up patients. These results raise important questions about the teaching, supervision, and assessment of medical students in the context of their teams’ schedules.

First, the role of the intern with students appears to be increasingly important in the setting of shift-based schedules. This largely reflects that, in the university schedule, interns do not rotate through swing and night shifts and, therefore, remain in more continuity with the medical students. Although perhaps less optimal than experienced clinician educators (2, 16, 17), house officers have been shown to have important roles in student career choice as well as undergraduate medical education (18–22). Our study suggests that additional time and resources should be invested in efforts to ensure that interns (or the resident level maximally paired with the medical student) are ready to serve as teachers. Additionally, these house officers are in a unique position to evaluate medical students and could provide an assessment of medical student performance based on direct observations. Furthermore, in our study, very few students felt that attendings were able to directly observe them at the patient's bedside. House officer training in medical student assessment will be critical, given this changing role.

Second, our results that shift-based schedules increase student exposure to previously worked-up patients are consistent with the literature to date (23). A prior study by Lang et al. suggested that teams admitting daily rather than on an every fourth day call cycle increased student exposure to hand-off patients by 50% (24). They found a small association between ‘fresh’ patients without prior evaluation seen by medical students and improved performance on the National Board of Medical Examiners (NBME) subject exam (24). Further analysis suggested that those students in the lowest quartile of performance on the NBME benefitted the most from admission of ‘fresh’ patients (24), suggesting that application of shift-based schedules adversely affects the performance of those students who are most at risk.

Third, exposure to the shift schedule decreases medical student perception of attendings’ commitment to teaching but not that of residents or interns. This may be due to increased attending physician stress in the setting of rapid team turnover. Additionally, changes in resident and intern schedules may have adversely affected attending physicians in other ways including increased perceived workload (25). These changes came at a time when stress and burnout among US physicians is already very high (26–28).

This study has several limitations. First, self-reported survey data are, by its very nature, subjective. The sample size was small and from a single institution, limiting generalizability. The low response rate introduces the possibility of non-response bias. Although students rotated through two of six possible sites, only a single survey was conducted at the end of their rotation, introducing recall bias and potentially introducing additional confounding variables not solely explained by exposure to shift-based schedules. However, faculty and site evaluations across sites were otherwise consistent for the clerkship as a whole on end of rotation evaluations. Moreover, given that we counted any exposure to the shift-based system as exposed, recall bias would likely reduce the impact of shift-based systems on overall student experiences. Also, although the results showed multiple statistical differences, the precise magnitude of these differences are difficult to determine. More exposed students came from Spring 2012 data due to the university adopting a shift-based, rotating resident schedule after duty-hour reform, potentially causing the data to reflect the impact of duty-hour changes.

## Summary/next steps

Despite these limitations, we believe that this is one of the first studies to assess the impact of shift-based schedules on medical students. Designing longitudinal team-based experiences could enhance relationships between students, residents, and attendings thereby improving team-based education and ultimately patient care (2). Continued research should be conducted to explore the impact of shift-based schedules and team discontinuity, and efforts towards limiting its effects should be implemented and evaluated.
